# ALDH2 protects naturally aged mouse retina *via* inhibiting oxidative stress-related apoptosis and enhancing unfolded protein response in endoplasmic reticulum

**DOI:** 10.18632/aging.202325

**Published:** 2020-12-19

**Authors:** Pan Long, Mengshan He, Weiming Yan, Wei Chen, Dongyu Wei, Siwang Wang, Zuoming Zhang, Wei Ge, Tao Chen

**Affiliations:** 1 Department of General Practice, Xijing Hospital, Fourth Military Medical University, Xi’an 710032, Shaanxi Province, China; 2 Department of Ophthalmology, The General Hospital of Western Theater Command, Chengdu 610083, Sichuan Province, China; 3 Department of Chinese Material Medical and Natural Medicines, Fourth Military Medical University, Xi’an 710032, Shaanxi Province, China; 4 Department of Ophthalmology, The 900th Hospital of Joint Logistic Support Force, PLA (Clinical Medical College of Fujian Medical University, Dongfang Hospital Affiliated to Xiamen University), Fuzhou 350025, Fujian Province, China; 5 Center of Clinical Aerospace Medicine, Fourth Military Medical University, Xi'an 710032, Shaanxi Province, China; 6 Department of Aviation Medicine, Xijing Hospital, Fourth Military Medical University, Xi’an 710032, Shaanxi Province, China; 7 Department of Pharmacy, Xijing Hospital, Fourth Military Medical University, Xi’an 710032, Shaanxi Province, China

**Keywords:** ALDH2, UPR^ER^, retina, aged mice, oxidative stress

## Abstract

During the process of aging, the retina exhibits chronic oxidative stress (OS) damage. Our preliminary experiment showed that acetaldehyde dehydrogenase 2 (ALDH2) could alleviate retinal damage caused by OS. This study aimed to explore whether ALDH2 could inhibit mice retinal cell apoptosis and enhance the function of unfolded protein response in endoplasmic reticulum (UPR^ER^) through reducing OS in aging process. Retinal function and structure *in vivo* and *in vitro* were examined in aged *ALDH2+* overexpression mice and ALDH2 agonist Alda1-treated aged mice. Levels of ALDH2, endoplasmic reticulum stress (ERS), apoptosis and inflammatory cytokines were evaluated. Higher expression of ALDH2 was observed at the outer nuclear layer (ONL) and the inner nuclear layer (INL) in aged *ALDH2+* overexpression and aged Alda1-treated mice. Moreover, aged *ALDH2+* overexpression mice and aged Alda1-treated mice exhibited better retinal function and structure. Increased expression of glucose-regulated protein 78 (GRP78) and ERS-related protein phosphorylated eukaryotic initiation factor 2 (peIF2α) and decreased expression of apoptosis-related protein, including C/EBP homologous protein (CHOP), caspase12 and caspase9, and retinal inflammatory cytokines were detected in the retina of aged *ALDH2+* overexpression mice and aged Alda1-treated mice. The expression of ALDH2 in the retina was decreased in aging process. ALDH2 could reduce retinal oxidative stress and apoptosis, strengthen UPR^ER^ during the aging process to improve retinal function and structure.

## INTRODUCTION

The aging process is characterized by a decline in systematic tissue function and the onset of serious of age-related disease [1–3]. However, it remained elusive that the potential mechanisms accounting for these phenomena and strategies to intervene to improve cell functions.

Recently, the imbalance of protein homeostasis was proposed to be responsible for aging and age-related diseases [4, 5]. As we known that the protein homeostasis is an essential component of the cell survival process and often refers to the maintenance of the proteome. And the unfolded protein response in the endoplasmic reticulum (UPR^ER^) is considered as the most well-known mechanisms of protein homeostasis. The endoplasmic reticulum (ER) is responsible for folding and quality control through the activation of chaperones and foldases that assist in protein folding and preventing abnormal protein aggregation [6]. The key proteins involved in UPR^ER^ include the sensors inositol requiring element-1 (IRE1), protein kinase R-like endoplasmic reticulum kinase (PERK) and activating transcription factor 6 (ATF6) and the molecular chaperone glucose-regulated protein 78 (GRP78), which is also called immunoglobulin heavy chain binding protein (BiP/GRP78). When superfluous unfolded or misfolded proteins accumulate in the ER, GRP78 disassociates from these three sensors, thus triggering the UPR^ER^ response [7]. In this time, GRP78, with this single existence, is easily encountered with oxidative stress damage, especially in aging process. Furthermore, eukaryotic translation initiation factor 2α (eIF2α), a protein translation component, is the downstream of PERK. When endoplasmic reticulum stress (ERS) happens, eIF2α is phosphorylated to slow down protein production and reduce the unfolded and misfolded proteins. [8]

During the aging process, protein glycosylation, oxidative imbalance, and protein folding defection cause abnormal protein accumulation in the ER and affect the UPR^ER^ to regulate the protein homeostasis capacity [9]. Moreover, increased free radicals damage during senescence leads to cell apoptosis via the caspase pathway, which is an important contributor to many human pathological conditions [10, 11]. Related studies have shown that the UPR^ER^ declined and its related chaperones were significantly reduced following with advancing age [12]. Traditionally, the UPR^ER^ is activated under the condition of energy deficiency with the aim of restoring protein homeostasis [13]. More importantly, the retina is one of the most energy-demanding tissues in the body. Both clinical and animal studies have found that retinal function and structure were worsen along with aging [14]. Nevertheless, the underlying mechanisms of UPR^ER^ related to aged retinal behaviour remain largely unclear, and effective therapies to intervene aging-related injury to the retina by targeting the UPR^ER^ have not been developed.

Mitochondrial aldehyde dehydrogenase 2(ALDH2) is essential for the catabolism of exogenous and endogenous toxic aldehydes associated with oxidative stress-induced lipid peroxidation and adducts with DNA, RNA and protein [15]. The available evidence suggested that chronic accumulation of aldehydes resulting from ALDH2 deficiency was responsible for the age-related diseases [16]. Recently, genetic polymorphism analysis of ALDH2 revealed that ALDH2*1*1 (wild homozygous GG) individuals had superior physical functions and diabetic or ischaemic stroke-related diseases resistance ability [17]. Interestingly, diabetic retinopathy (DR) was also closely related to ALDH2 polymorphisms [18, 19]. Moreover, ALDH2 has a certain protective effect in age-related disease, such as Alzheimer's Disease (AD), Parkinson's Disease (PD) and aged cardiopathy by decreasing free radicals damage [20]. Simultaneously, our previous animal experiments have also shown that ALDH2 could play a certain role in aged diabetic retinopathy [21] and retinitis pigmentosa (RP) [22]. Furthermore, our clinical retrospective data analysis showed that the aged with ALDH2*1* (G/G) genotype possessed a better retinal function and a thicker macula compared with ALDH2*2*2 (mutant heterozygous AA) while the young with different ALDH2 genotype, including ALDH2*1*1, ALDH2*1*2 (mutant heterozygous GA) and ALDH2*2*2, showed no significant difference (Supplementary Figure 1).

Considering that ALDH2 could alleviate OS damage, ALDH2 overexpression in aged mice (transgenic mice controlled by EF1α and chicken β-actin promoters) and aged mice treated with the ALDH2 activator Alda1 (N-[1,3-benzodioxol-5-ylmethyl]-2,6-dichlorobenzamide) were examined to evaluate the effect of ALDH2 on aged mice retinal function and structure, and explore the underlying mechanisms.

## RESULTS

### Expression of ALDH2 in different groups

**Figure 1:**
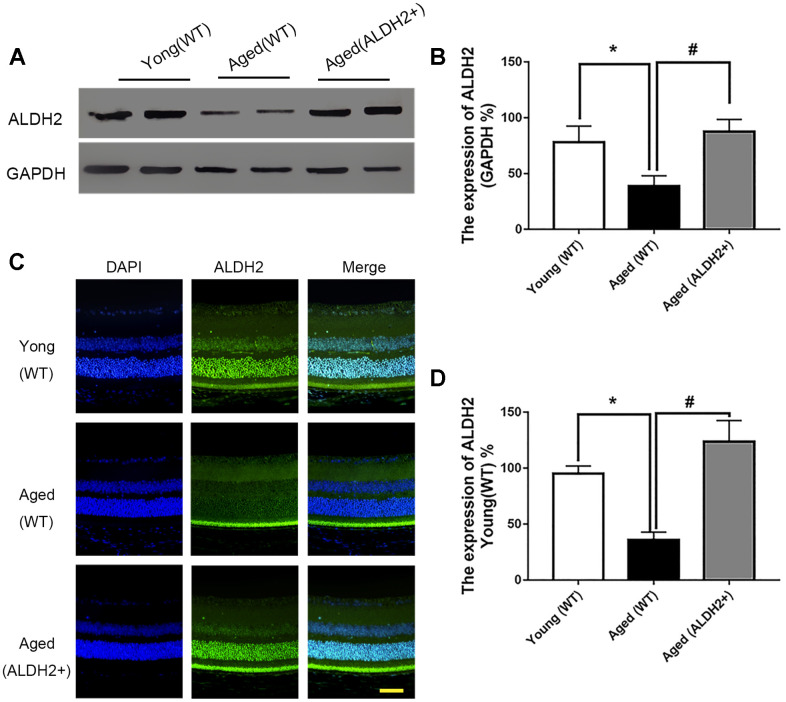
**The expression of ALDH2 in the young (WT) group, aged (WT) group and aged (ALDH2+) group.** (**A**, **B**) A typical ALDH2 WB image and the expression of ALDH2; (**C**, **D**) A typical ALDH2 immunofluorescence image and the expression of ALDH2. All analyses were performed in duplicate. Scale bar: 50 μm. Values are presented as the mean ± SD, n = 4 mice per group. *P<0.05: aged (WT) group and aged (ALDH2+) group *vs* young (WT) group; ^#^*P*<0.05: aged (ALDH2+) group *vs* aged (WT) group.

**Figure 2:**
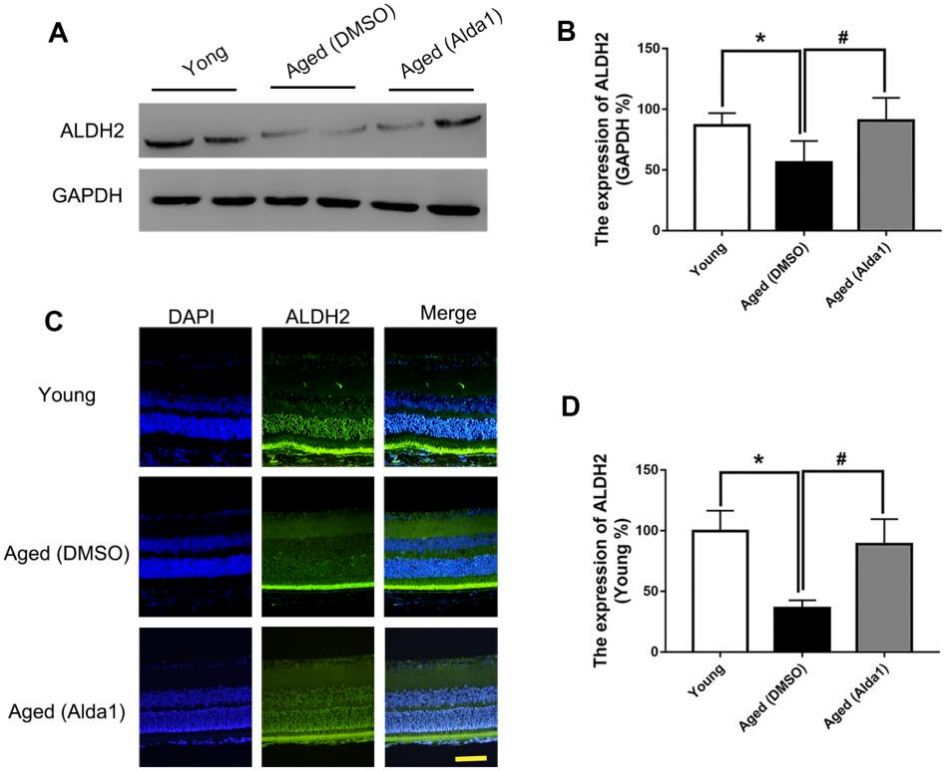
**The expression of ALDH2 in the young group, aged (DMSO) group and aged (Alda1) group.** (**A**, **B**) A typical ALDH2 WB image and the expression of ALDH2; (**C**, **D**) A typical ALDH2 immunofluorescence image and the expression of ALDH2. All analyses were performed in duplicate. Scale bar: 50 μm. Values are presented as the mean ± SD, n = 4 mice per group. *P<0.05: aged (DMSO) group and aged (Alda1) group *vs* young group; ^#^*P*<0.05: aged (Alda1) group *vs* aged (DMSO) group.

Total retinal proteins detected by western blotting (WB) showed that the expression of ALDH2 was dramatically decreased in the aged (WT) group compared with the young (WT) group (*P*<0.05). Furthermore, ALDH2 expression was significantly increased in the aged (ALDH2+) group and aged (Alda1) group compared with the aged (WT) group and aged (DMSO) group, respectively (all *P*<0.05) (Figure 1A, 1B, Figure 2A, 2B). Moreover, as shown in Figure 1C, 1D and Figure 2C, 2D, immunofluorescence staining of retinal paraffin sections from the young (WT) group showed higher ALDH2 expression in the ONL and INL of the retinas. In contrast, retinas from the aged (WT) group exhibited lower ALDH2 expression in the ONL and INL. Interestingly, the retinas from the aged (ALDH2+) group and aged (Alda1) group showed obvious enhancing expression of ALDH2 in the ONL and INL compared with the aged (WT) group and aged (DMSO) group, respectively (all *P*<0.05). Thus, the immunofluorescence staining and western blotting results were consistent.

### ALDH2 improved aged mouse total retinal function

To evaluate the potential role of ALDH2 in retinal function, electroretinography (ERG) detection was performed. The value of the b wave (dark-adaptation 3.0 response) was selected to evaluate the mice total retinal function as the dark-adaptation 3.0 response is related to both cone cell and rod cell functions.

**Figure 3:**
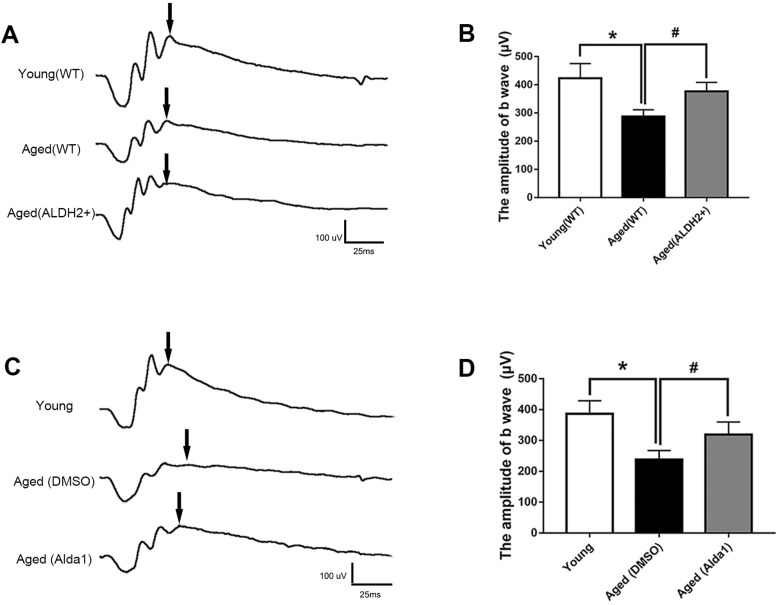
**ALDH2 enhanced aged mouse retinal function.** (**A**, **B**) A typical dark-adaptation 3.0 response image and amplification of the dark-adaptation 3.0 response b wave in ALDH2 overexpression mice; (**C**, **D**) A typical dark-adaptation 3.0 response image and the amplification of the dark-adaptation 3.0 response b wave in Alda1-treated mice. All analyses were performed in duplicate. Values are presented as the mean ± SD, n = 10 mice per group. *P<0.05: aged (WT) group and aged (ALDH2+) group *vs* young (WT) group or aged (DMSO) group and aged (Alda1) group *vs* young group; ^#^*P*<0.05: aged (ALDH2+) group *vs* aged (WT) group or aged (Alda1) group *vs* aged (DMSO) group.

As shown in Figure 3A, 3B, the value of the b wave (dark-adaptation 3.0 response) was significantly decreased in the aged (WT) group compared with the young (WT) group (*P*<0.05). However, the value of the b wave (dark-adaptation 3.0 response) was increased in the aged (ALDH2+) group compared with the aged (WT) group (*P*<0.05), while there was no significant difference between the aged (ALDH2+) group and young (WT) group (*P*>0.05). Moreover, as shown in Figure 3C, 3D, the value of the b wave (dark-adaptation 3.0 response) was significantly decreased in the aged (DMSO) group compared with the young group (*P*<0.05), and the value of the b wave (dark-adaptation 3.0 response) was increased in the aged (Alda1) group compared with the aged (DMSO) group (*P*<0.05). There were no significant differences between the aged (Alda1) group and young group (*P*>0.05).

### ALDH2 enhanced aged mouse vessel function

**Figure 4:**
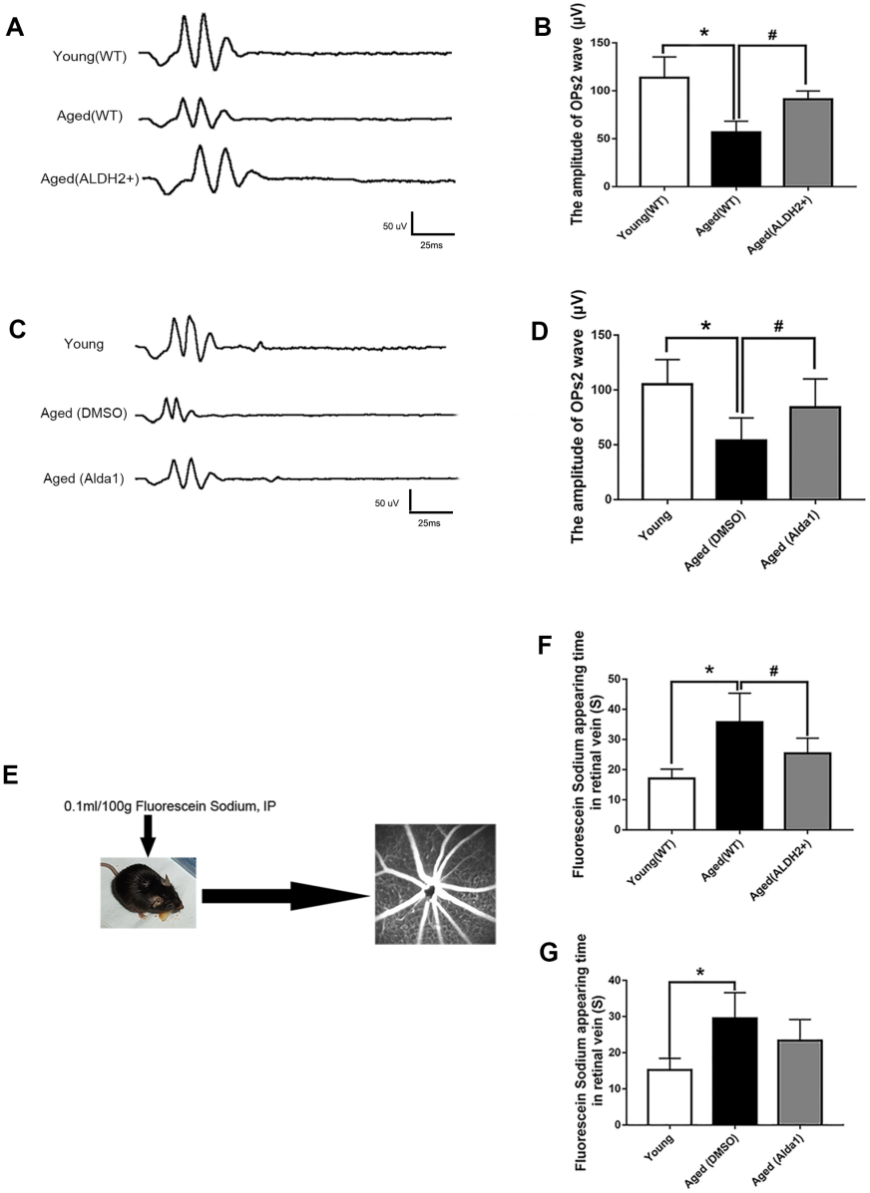
**ALDH2 enhanced aged mouse retinal vessel function.** (**A**, **B**) A typical OPs response image and amplification of the OPs2 response in ALDH2 overexpression mice; (**C**, **D**) A typical OPs response image and the amplification of OPs2 response in Alda1-treated mice; (**E**) The operation procedure pattern diagram for the FFA detection method; (**F**) The appearance time of fluorescein sodium in the retinal vessel in ALDH2 overexpression mice (**G**) and Alda1-treated mice. All analyses were performed in duplicate. Values are presented as the mean ± SD, n = 10 mice per group. *P<0.05: aged (WT) group and aged (ALDH2+) group *vs* young (WT) group or aged (DMSO) group and aged (Alda1) group *vs* Young group; ^#^ *P*<0.05: aged (ALDH2+) group *vs* aged (WT) group or aged (Alda1) group *vs* aged (DMSO) group.

To explore the retinal vessel protective effect of ALDH2, retinal vessel related ERG response (oscillatory potentials, OPs response) and FFA detection (the operation procedure pattern diagram was showed in Figure 4E) were performed. The OPs wave, as an ERG component, could be used to indirectly evaluate retinal vessel function, because retinal amacrine cells are accompanied by retinal vessels.

The results showed that the OPs2 wave (a typical wave of OPs) value was significantly decreased in the aged (WT) group compared with the young (WT) group (*P*<0.05) (Figure 4A, 4B). Moreover, the value of the OPs2 wave was increased in the aged (ALDH2+) group compared with the aged (WT) group (*P*<0.05), while there was no significant difference compared with the young (WT) group (*P*>0.05) (Figure 4A, 4B). In addition, the OPs2 wave value was significantly decreased in the aged (DMSO) group compared with the young group (*P*<0.05). Moreover, the OPs2 wave value was increased in the aged (Alda1) group compared with the aged (DMSO) group (*P*<0.05), while there was no significant difference compared with the young group (*P*>0.05) (Figure 4C, 4D).

Furthermore, we calculated the time of appearance of fluorescein sodium in the retinal vein following the first intraperitoneal injection of fluorescein sodium to evaluate the function of the retinal vessel microcirculation. As shown in Figure 4F, 4G, the time of appearance of fluorescein sodium was significantly prolonged in the aged (WT) group compared with the young (WT) group (*P*<0.05). In the aged (ALDH2+) group, the time of appearance of fluorescein sodium was significantly reduced compared with the aged (WT) group (*P*<0.05). In the aged (DMSO) group, the time of appearance of fluorescein sodium was significantly prolonged compared with the young group (*P*<0.05). However, the time of appearance of fluorescein sodium showed no significant difference in the aged (Alda1) group compared with the aged (DMSO) group (*P*>0.05).

### ALDH2 protected the aged mouse retinal structure

**Figure 5:**
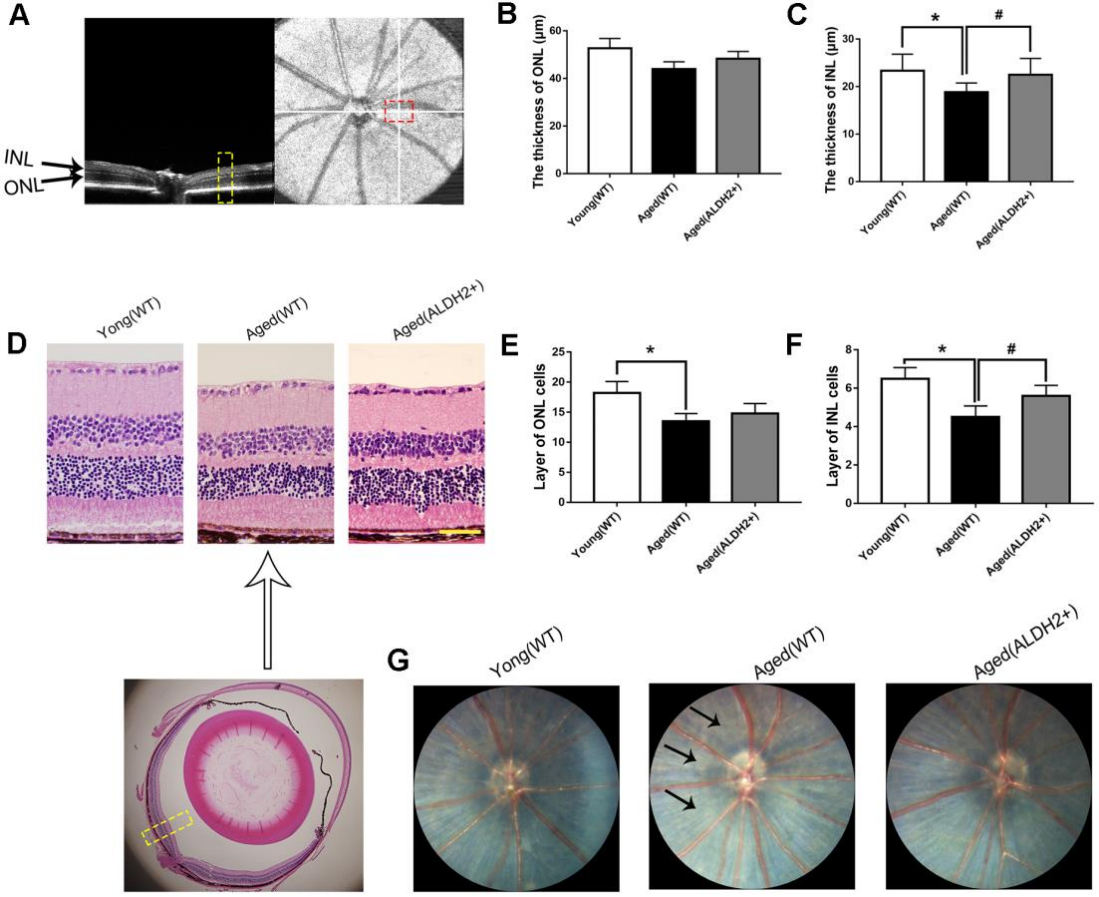
**The retinal structures of ALDH2 overexpression aged mice.** (**A**) A typical marked OCT image; (**B**) The thickness of the ONL; (**C**) The thickness of the INL; (**D**) A typical marked HE staining image; (**E**) The number of ONL cell layers; (**F**) The number of INL cell layers; (**G**) The fundus performance in the mice. All analyses were performed in duplicate. Scale bar: 50 μm. Black arrow showed retinal depigmentation change. Yellow box showed retinal structure analysis area. Values are presented as the mean ± SD, n = 6 mice per group. *P<0.05: aged (WT) and aged (ALDH2+) *vs* young (WT); ^#^*P*<0.05: aged (ALDH2+) *vs* aged (WT).

To further examine the protective roles of ALDH2 in the aged mouse retinal structure, optical coherence tomography (OCT) and Hematoxylin-Eosin (HE) staining were performed. OCT could determine every retinal layer according to the different luminance reflective bands, such as the low-reflective band representative of ONL and INL and the high-reflective band representative of the retinal nerve fibre layer (RNFL), inner plexiform layer (IPL), outer plexiform layer (OPL), photoreceptor inner segment/outer segment (IS/OS) junction line and retinal pigment epithelium (RPE) layer (Figure 5A).

As shown in Figure 5B, 5C, the thickness of the INL detected by OCT was significantly decreased in the aged (WT) group compared with the young (WT) group (*P*<0.05). Interestingly, the thickness of INL was increased in the aged (ALDH2+) group compared with the aged (WT) group (*P*<0.05). However, there were no significant differences in the ONL thickness among each group (*P*>0.05). As shown in Figure 5D–5F, HE staining showed that the ONL cell and INL cell layers were significantly reduced in the aged (WT) group compared with those in the young (WT) group (all *P*<0.05). Furthermore, the INL cell layer was more in the aged (ALDH2+) group than the aged (WT) group (*P*<0.05). Moreover, the fundus photograph revealed a depigmentation change in the aged (WT) group but little depigmentation change in the aged (ALDH2+) group (Figure 5G).

**Figure 6:**
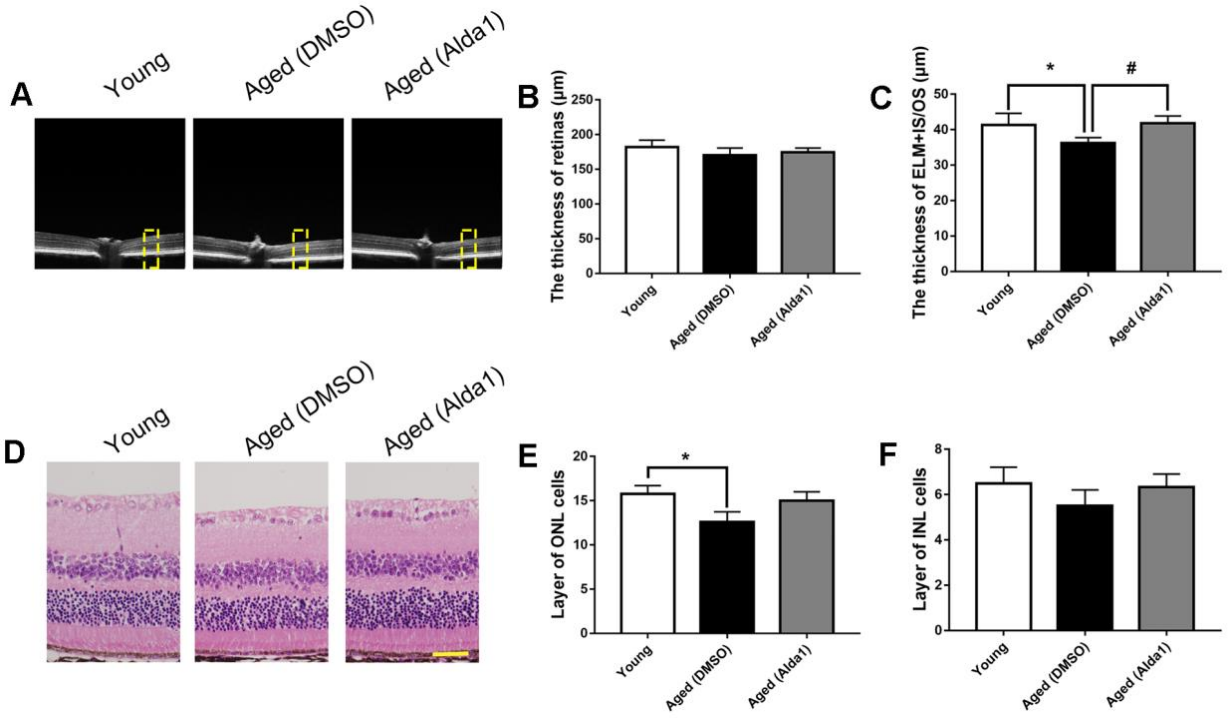
**The retinal structures in Alda1-treated aged mice.** (**A**) A typical marked OCT image; (**B**) The thickness of the total retina; (**C**) The thickness of the ELM+IS/OS. (**D**) A typical marked HE staining image; (**E**) The number of ONL cell layers; (**F**) The number of INL cell layers. All analyses were performed in duplicate. Scale bar: 50 μm. Yellow box showed retinal structure analysis area. Values are presented as the mean ± SD, n = 6 mice per group. *P<0.05: aged (WT) and aged (ALDH2+) *vs* young (WT); ^#^*P*<0.05: aged (ALDH2+) *vs* aged (WT).

Regarding the ALDH2 agonist, the total retinal thickness among the young, aged (DMSO) and aged (Alda1) groups was not significantly different (all *P*>0.05). However, the thickness of ELM+IS/OS was reduced in the aged (DMSO) group compared with the young group (*P*<0.05), while it was increased in the aged (Alda1) group compared with that in the aged (DMSO) group (*P*<0.05) (Figure 6A–6C). Moreover, HE staining showed that the number of INL cell layers was significantly reduced in the aged (DMSO) group compared with the young group (*P*<0.05). For the number of ONL cell layers, there existed no significant difference among the groups (Figure 6D–6F).

### ALDH2 decreased the expression of TNF-α, IL-6 and IL-1 in aged mouse retina

**Figure 7:**
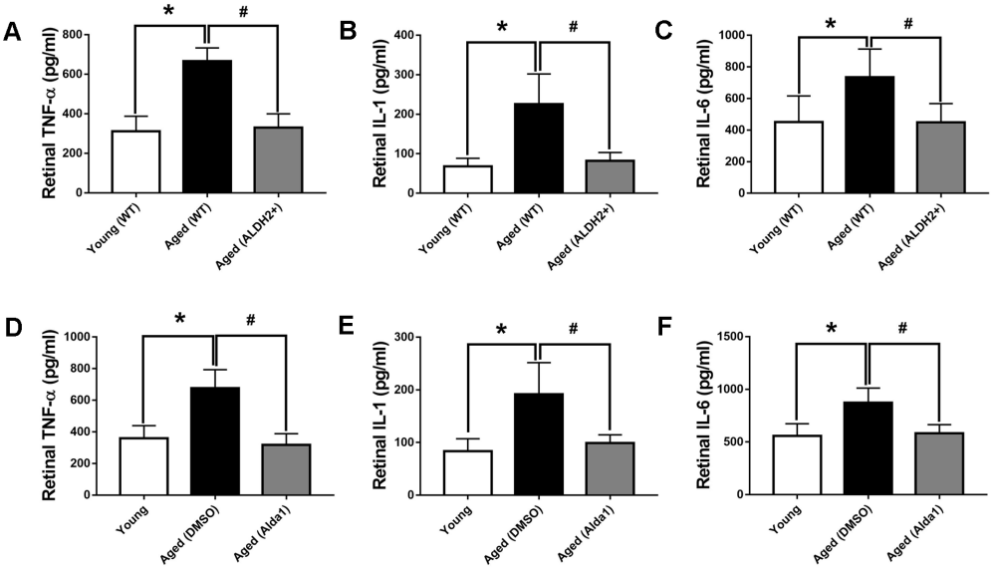
**The expression of retinal TNF-α, IL-6 and IL-1 in aged ALDH2 overexpression and aged Alda1-treated mice.** (**A**) The expression of retinal TNF-α in aged ALDH2 overexpression mice; (**B**) The expression of retinal IL-6 in aged ALDH2 overexpression mice; (**C**) The expression of retinal IL-1 in aged ALDH2 overexpression mice; (**D**) The expression of retinal TNF-α in aged Alda1-treated mice; (**E**) The expression of retinal IL-6 in aged Alda1-treated mice; (**F**) The expression of retinal IL-1 in aged Alda1-treated mice. All analyses were performed in duplicate. Values are presented as the mean ± SD, n = 4 mice per group. *P<0.05: aged (WT) and aged (ALDH2+) *vs* young (WT) or Aged (DMSO) and aged (Alda1) *vs* young; ^#^*P*<0.05: aged (ALDH2+) *vs* aged (WT) or aged (Alda1) *vs* aged (DMSO).

In aging process, oxidation imbalance, accompanied by free radicals accumulation, affects retina working regularly. Retinal inflammatory factor, including tumor necrosis factor-α (TNF-α), interleukin-6 (IL-6) and interleukin-1 (IL-1) were detected to evaluate oxidative stress damage with aging. As shown in Figure 7A–7C, the expression levels of retinal TNF-α, IL-6 and IL-1 were significantly increased in the aged (WT) group compared with those in the young (WT) group, respectively (all *P*<0.05). However, the expression of retinal TNF-α, IL-6 and IL-1 were decreased in the aged (ALDH2+) group compared with those in the aged (WT) group, respectively (all *P*<0.05). As for the agonist intervention experiment, the expression of retinal TNF-α, IL-6 and IL-1 were increased in the aged (DMSO) group compared with those in the young group, respectively (all *P*<0.05). Additionally, the expression of retinal TNF-α, IL-6 and IL-1 were decreased in the aged (Alda1) group compared with those in the aged (DMSO) group, respectively (all *P*<0.05) (Figure 7D–7F).

### ALDH2 enhanced UPR^ER^ in aged mouse retina

**Figure 8:**
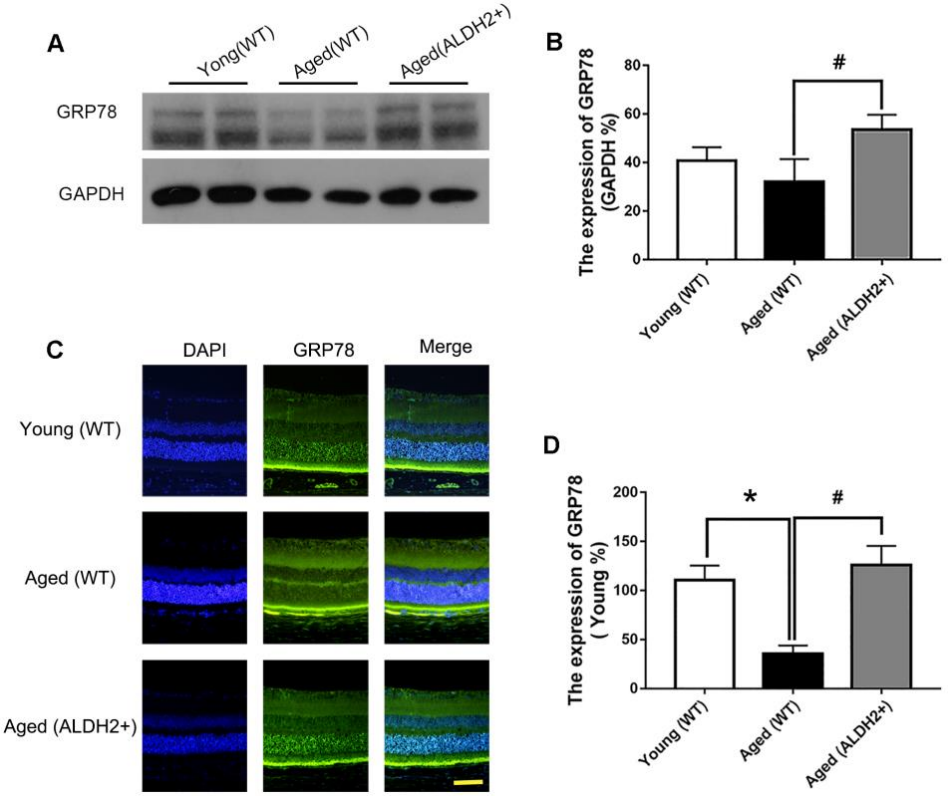
**The expression of GRP78 in ALDH2+ overexpression mice during the normal aging process.** (**A**, **B**) A typical WB image of GRP78 and the confirmation of GRP78 expression by WB; (**C**, **D**) A typical immunofluorescence image of GRP78 and the confirmation of GRP78 expression by immunofluorescence. All analyses were performed in duplicate. Scale bar: 50 μm. Values are presented as the mean ± SD, n = 4 mice per group. *P<0.05: aged (WT) and aged (ALDH2+) *vs* young (WT); ^#^*P*<0.05: aged (ALDH2+) *vs* aged (WT).

To determine whether ALDH2 was involved in the enhancement of UPR^ER^ during the retinal aging process, the retinal UPR^ER^-related proteins GRP78, eIF2α and phosphorylated eIF2α (peIF2α) were assessed. As shown in Figure 8A, 8B, GRP78 expression was decreased in the aged (WT) group compared with the young (WT) group, as determined by western blotting (*P*<0.05). Additionally, GRP78 expression was increased in the aged (ALDH2+) group compared with the aged (WT) group, as confirmed by western blotting analysis (*P*<0.05). Immunofluorescence staining showed that GRP78 was highly expressed in ONL, which was consistent with the western blotting results (Figure 8C, 8D).

**Figure 9:**
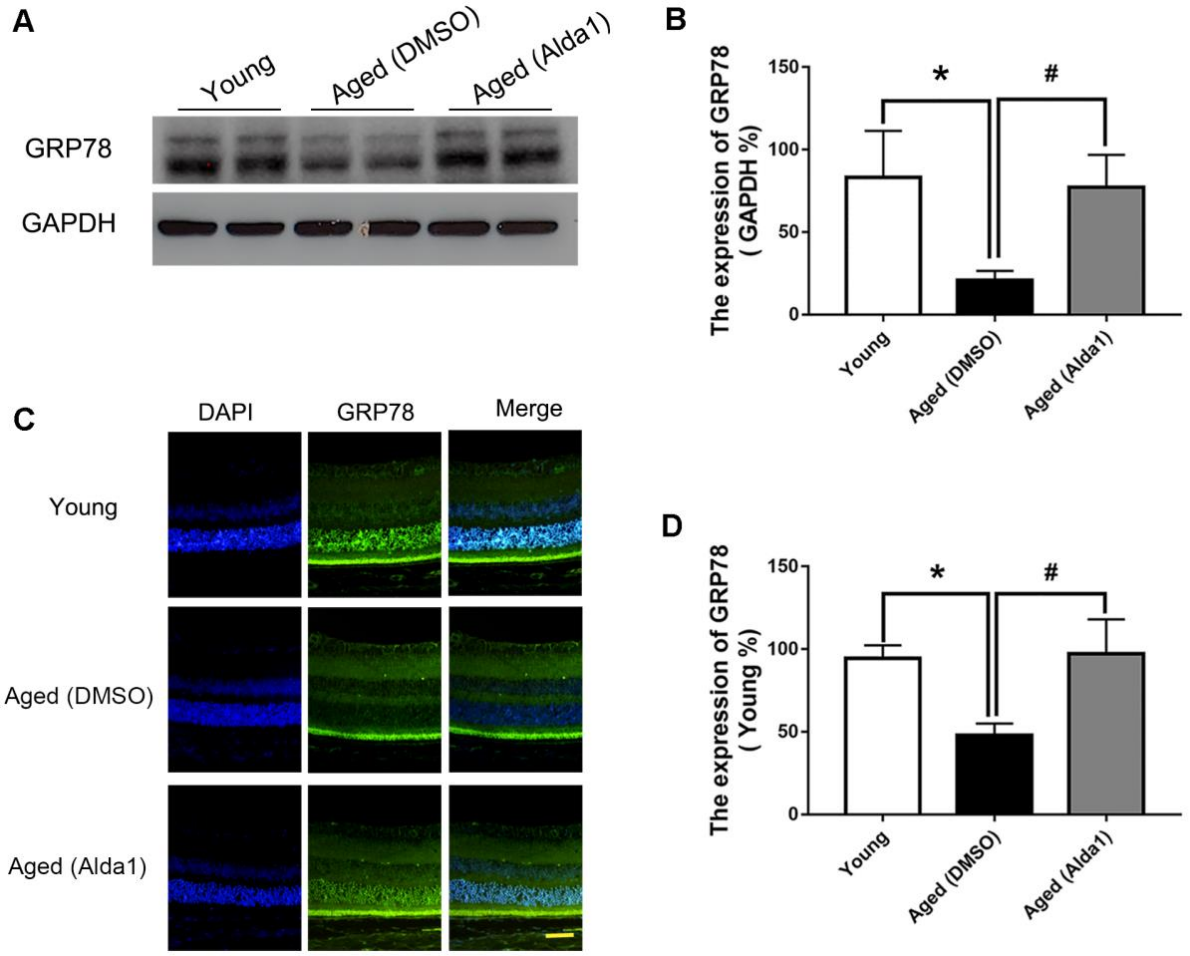
**The expression of GRP78 in Alda1-treated mice during the normal aging process.** (**A**, **B**) A typical WB image of GRP78 and the confirmation of GRP78 expression by WB; (**C**, **D**) A typical immunofluorescence image of GRP78 and the confirmation of GRP78 expression by immunofluorescence. Scale bar: 50 μm. All analyses were performed in duplicate. Values are presented as the mean ± SD, n = 4 mice per group. *P<0.05: aged (DMSO) and aged (Alda1) *vs* young; ^#^*P*<0.05: aged (Alda1) *vs* aged (DMSO).

Additionally, western blotting (Figure 9A, 9B) and immunofluorescence staining (Figure 9C, 9D) results showed that the expression of GRP78 was dramatically decreased in the aged (DMSO) group compared with that in the young group (*P*<0.05), while the expression of GRP78 was dramatically increased in the aged (Alda1) group compared with the aged (DMSO) group (*P*<0.05).

**Figure 10:**
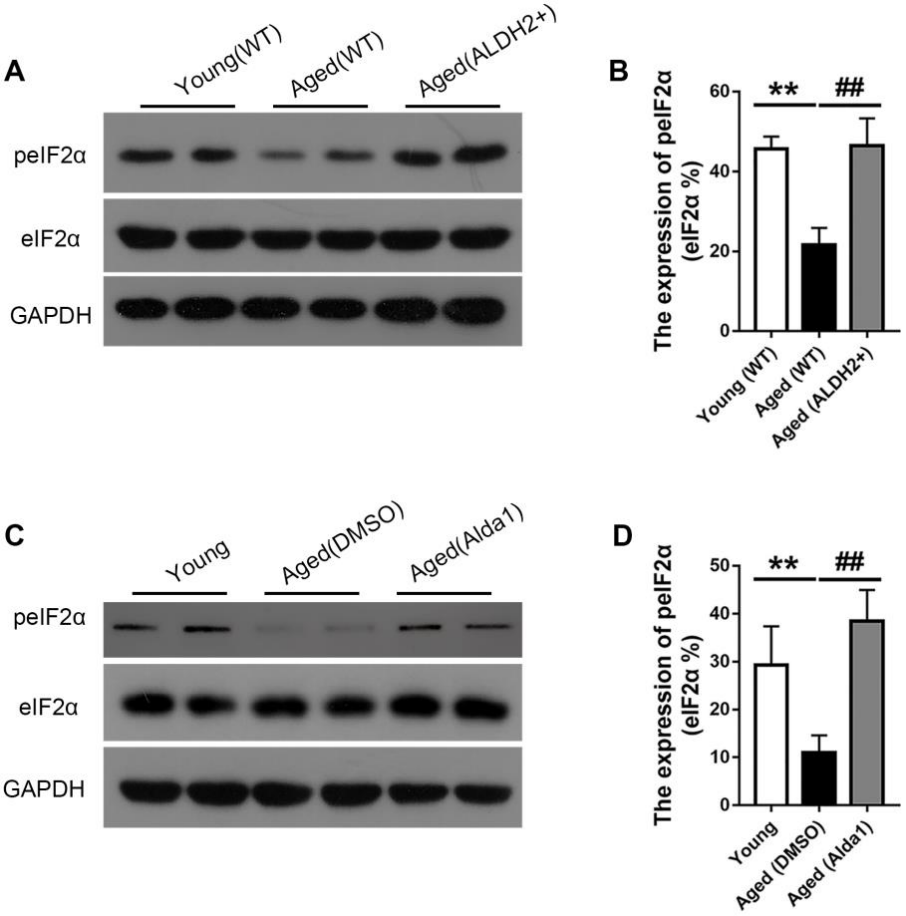
**The expression of eIF2α and peIF2α in aged ALDH2 overexpression mice and aged Alda1-treated mice.** (**A**) A typical WB image of eIF2α and peIF2α in aged ALDH2 overexpression mice; (**B**) The expression of peIF2α/eIF2α in aged ALDH2 overexpression mice; (**C**) a typical WB image of eIF2α and peIF2α in aged Alda1-treated mice; (**D**) The expression of peIF2α/eIF2α in aged Alda1-treated mice. All analyses were performed in duplicate. Values are presented as the mean ± SD, n = 4 mice per group. **P<0.01: aged (WT) group and aged (ALDH2+) group *vs* young (WT) group or aged (DMSO) group and aged (Alda1) group *vs* Young group; ^##^*P*<0.01: aged (ALDH2+) group *vs* aged (WT) group or aged (Alda1) group *vs* aged (DMSO) group.

Moreover, the expression of peIF2α was obviously reduced in the aged (WT) group compared with that in the young (WT) group (*P*<0.05), while the expression of peIF2α was increased in the aged (ALDH2+) group compared with that in the aged (WT) group (*P*<0.05) (Figure 10A, 10B). Furthermore, the expression of peIF2α was decreased in the aged (DMSO) group compared with that in the young group (*P*<0.05), while its expression was increased in the aged (Alda1) group compared with that in the aged (DMSO) group (*P*<0.05) (Figure 10C, 10D).

### ALDH2 decreased apoptosis-related protein in aged mouse retina

**Figure 11:**
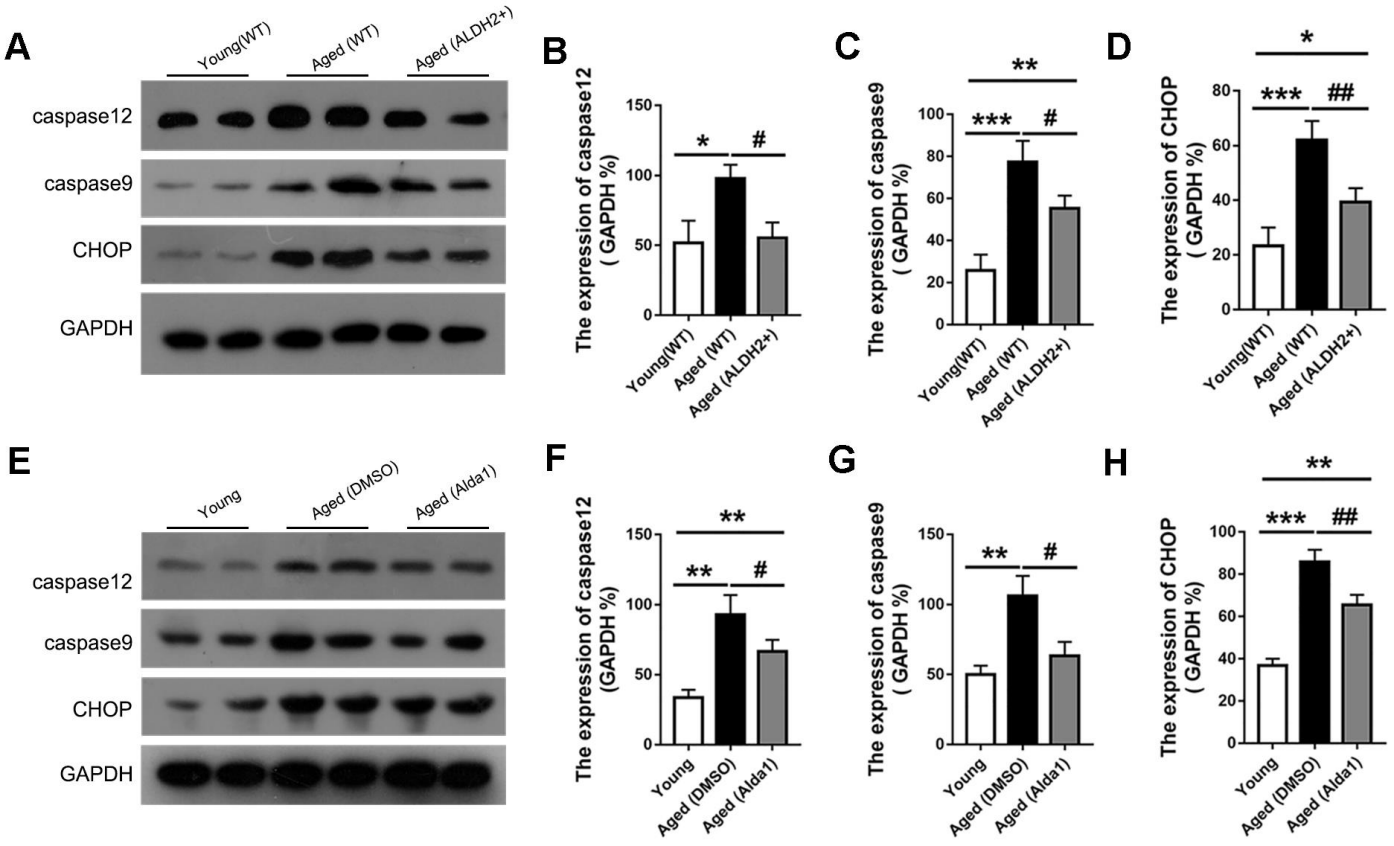
**The expression of apoptosis related proteins in aged ALDH2 overexpression and aged Alda1-treated mice.** (**A**) A typical WB image of CHOP, caspase9 and caspase12 in aged ALDH2 overexpression mice; (**B**–**D**) The expression of CHOP, caspase9 and caspase12 in aged ALDH2 overexpression mice; (**E**) A typical WB picture of CHOP, caspase9 and caspase12 in aged Alda1-treated mice; (**F**–**H**) The expression of CHOP, caspase9 and caspase12 in aged Alda1-treated mice. All analyses were performed in duplicate. Values are presented as the mean ± SD, n = 4 mice per group. *P<0.05: aged (WT) and aged (ALDH2+) *vs* young (WT) or Aged (DMSO) and aged (Alda1) *vs* young; **P<0.01: aged (WT) and aged (ALDH2+) *vs* young (WT) or Aged (DMSO) and aged (Alda1) *vs* young; ***P<0.001: aged (WT) and aged (ALDH2+) *vs* young (WT) or Aged (DMSO) and aged (Alda1) *vs* young; ^#^*P*<0.05: aged (ALDH2+) *vs* aged (WT) or aged (Alda1) *vs* aged (DMSO), ^##^*P*<0.01: aged (ALDH2+) *vs* aged (WT) or aged (Alda1) *vs* aged (DMSO).

Apoptosis was accompanied with aging process, and we detected the proapoptotic protein CHOP and apoptosis protein caspase12 and caspase9. As shown in Figure 11A–11D, the expression of CHOP, caspase12 and caspase9 were significantly increased in the aged (WT) group compared with those in the young (WT) group (all *P*<0.05). However, the expression of CHOP, caspase12 and caspase9 were decreased in the aged (ALDH2+) group compared with the aged (WT) group (all *P*<0.05). Moreover, the expression of caspase12 existed no significant difference between the young (WT) group and aged (ALDH2+) group (*P*>0.05).

Regarding the agonist intervention experiment, the expression levels of CHOP, caspase12 and caspase9 were increased in the aged (DMSO) group compared with those in the young group (all *P*<0.05). Additionally, the expressions of CHOP, caspase12 and caspase9 was with those in the young group (all *P*<0.05). Additionally, the expressions of CHOP, caspase12 and caspase9 was decreased in the aged (Alda1) group compared with those in the aged (DMSO) group (all *P*<0.05) (Figure 11E–11H).

## DISCUSSION

The aging process is accompanied by the hypofunction of retinal cells, and specific interventions that could preserve retinal function are highly desired. Here we confirmed a reduction of retinal function and structure during the natural aging process. More importantly, we found that ALDH2 could effectively improve the aged mice retina, and the potential mechanism might be related with a decreased oxidative stress induced apoptosis activity and an enhanced UPR^ER^.

Recent studies have explored the reduction of retinal function and structure in clinical and animal studies during the aging process. Specifically, clinical studies found a prolonged implicit time and declining OPs and b wave (dark-adaptation 3.0 response) amplitudes by 40 years of age [23]. Compared with the younger group, the b wave (dark-adaptation 3.0 response) amplitudes showed 25-40% smaller and longer implicit times [24]. Moreover, the bleaching restoration (dark adaptation ability) of amplitude was slower in older subjects [25]. Moreover, a recent study found that retinal thickness was reduced by 0.24 μm for every year with aging [26]. In a study of aging mice, it was found that aging disease-free retina underwent a reduction of photoreceptor density and ERG amplitudes [27]. It was found that aging was associated with a decrease in thickness of the photoreceptor layer [28]. Additionally, a reduction in the retinal nerve fibre layer (RNFL) thickness were correlated with increasing age [29].

During the aging process, functional protein, DNA and RNA, suffer from accumulated chronic oxidative damage, leading to mitochondrial dysfunction, cell apoptosis and endoplasmic reticulum dysfunction. As we known the endoplasmic reticulum (ER) could play essential roles in folding and transporting protein. When faced with unfolded or misfolded proteins, endoplasmic reticulum chaperone proteins, especially BiP/GRP78, would initiate correct protein folding. Recent studies have found a decrease of UPR^ER^-related proteins in the patients with aging diseases [30]. These ER chaperones encounter with oxidative damage, which may limit their ability to perform appropriate protein folding and therefore reduce the ability of the UP^RER^ to regulate protein homeostasis [31, 32]. Among the ER stress pathways, GRP78 is a master regulator of the UPR^ER^ by handling abnormal proteins to recover ER homeostasis [33]. Therefore, the aging process is associated with a prominent reduction in the buffering capacity of the protein homeostasis network [34]. Moreover, studies have confirmed that the ability to activate the UPR^ER^ declined along with aging, while its constitutive activation could extend longevity [35]. For retinas, the activation of UPR^ER^ is a protective response to abnormal protein accumulation that ultimately restricts photoreceptor cell death [36]. And overexpression of GRP78 could regulate the UPR^ER^ and preserve photoreceptor function [37]. Moreover, increasing expression of GRP78 could downregulate the key pro-apoptotic cascade activator CHOP [38].

Our studies showed that the UPR^ER^ remained deficient for successful disposing of abnormal proteins during the aging process. Related studies have shown that wild type aged mouse retinas showed a significant reduction basal level of X-box Binding Protein 1 spliced (XBP1s), a key ERS signalling pathway downstream protein, and decreased activation of the UP^RER^. Moreover, XBP1s KO mice showed significant retinal layer thinning and retinal ganglion cell (RGC) loss, as well as functional defects at 12-14 months of age [39]. To our knowledge, the UP^RER^ is characterized by acute UP^RER^ induced by hypoxia, nutrient deprivation, increased protein oxidation, and a disturbance of the secretory pathway, and chronic UP^RER^ induced especially by the senescence process. Acute UP^RER^, with stronger and uncontrolled features, could lead to cell death, while chronic UP^RER^, with limited and insufficient features, could also trigger cell apoptosis through the CHOP and caspase12 pathways [35]. Furthermore, metabolism studies have shown that aging mice have diminished ERG responses and a reduced number of photoreceptors [40].

In this study, ALDH2 could effectively prevent the decline in retinal function and structure damage, as confirmed both by ALDH2 overexpression mice and intervention with the ALDH2 agonist Alda1. It is well-known that ALDH2 possesses potential roles of protecting cellular bioactive molecules, such as DNA, RNA and protein, from the damage of toxic aldehydes. Therefore, ALDH2 could decrease the production of unfolded or misfolded protein. As a result, in different stages of life or diverse diseases, the key roles of ALDH2 could differ during their involvement in the UPR^ER^ process. In the early stage of life, ALDH2 mainly sustains cell protein homeostasis by reducing unfolded or misfolded protein production, and thus the activation of ALDH2 could decelerate the progression of diseases such as atherosclerosis via decreasing ERS and apoptosis [41]. Recent studies have revealed that ALDH2 overexpression could effectively antagonize chronic alcohol intake-induced cardiac injury and contractile defects via decreasing ERS-related cell apoptosis [42]. In contrast, ALDH2 deficiency aggravates cardiac dysfunction with an accumulation of abnormal protein, leading to an increase in related cell apoptosis [43]. However, during the aging process, ALDH2 could enhance the UPR^ER^ to improve the ability to correct the activity of unfolded or misfolded protein. Our study showed that ALDH2 effectively preserved the UPR^ER^ by protecting the molecular chaperone GRP78 away from the sustained oxidative stress injury, thus improving cell survival ability. Unfortunately, the accurate regulatory mechanisms of ALDH2 in the aging process are still unclear. We could be certain that during the aging process, the expression levels of retinal CHOP, caspase12 and caspase9 were increasing and retinal inflammatory factor, including IL-1, IL-6 and TNF-α expression were enhanced. And ALDH2 could attenuate those hazardous substances expression, therefore alleviate aging retina oxidative damage and free radical attack by eliminating oxidizing substances, especially organic peroxides. The potential aging retina protective effect of ALDH2 could include: 1. Participate in sustaining mitochondria homeostasis to reduce the production of peroxide; 2. Regulate nuclear gene expression (our additional data showed ALDH2 could interact with histone deacetylase); 3. Influence cell stress response in ER or ribosome.

In the present study, overexpression of ALDH2 and treatment with the ALDH2 agonist Alda1 in aging mice could both result in good retinal function and structural integrity via attenuating oxidative stress and apoptosis, and enhancing UPR^ER^. Therefore, an increasing expression of ALDH2 could serve to preserve retinal function during the normal aging process or the onset of age-related retinal disease.

## Materials and Methods

### Animals

Healthy male young C57/B6 mice (6-8 weeks old) made up the young (WT) group. Healthy male aged C57/B6 mice (72-80 weeks old) made up the aged (WT) group. Healthy male aged C57/B6 mice (72-80 weeks old) and aged *ALDH2+* C57/B6 mice (72-80 weeks old) made up the aged (WT) group and aged (ALDH2+) group, respectively. These mice were selected to directly examine retinal function and structure *in vivo* and ERS, apoptosis proteins and inflammatory factor *in vitro*. Furthermore, mice (48-56 weeks old) were subjected to (N-[1,3-benzodioxol-5-ylmethyl]-2,6-dichlorobenzamide) Alda1(Molecular Weight: 324.16; Formula: C_15_H_11_Cl_2_NO_3_) (MedChemExpress, USA) (10 mg/kg, 50% DMSO: 50% H_2_O) [44] or DMSO, as the aged (Alda1) group and the aged (DMSO) group, via daily intraperitoneal injection for 24 weeks. Moreover, young C57/B6 mice (6-8 weeks old) served as the young group. After the last treatment, retinal function and structures *in vivo* and ERS, apoptosis proteins and inflammatory factor *in vitro* were examined. Young mice were purchased from the Laboratory Animal Center of the Fourth Military Medical University (license: 2014270138S). Aged mice were purchased from Beijing Vital River Laboratory Animal Technology Co., Ltd. (license: SCXK2016-0006). ALDH2 overexpression C57/B6 mice (transgenic mice controlled by EF1α and chicken β-actin promoters) were obtained from professor Ren Jun (Center for Cardiovascular Research and Alternative Medicine, School of Pharmacy, University of Wyoming College of Health Sciences).

All animals were raised under clean laboratory conditions (temperature: 23° C ± 3° C; 12-h light/ 12-h dark cycles). All animals in the experiments were handled in accordance with the Association for Research in Vision and Ophthalmology (ARVO) Statements for the use of Animals in Ophthalmic and Vision Research. All protocols were approved by the research ethics committee for the care of laboratory animals at Fourth Military Medical University, and the experiments were conducted in accordance with its guidelines for experimental animals.

### Electroretinography

ERG measurements were performed as described previously [45]. Briefly, the animals were placed in a dark environment for 12 hours, and all operation processes were recorded under dim red-light conditions. Mice were anaesthetized with 3 mL/kg 1% sodium pentobarbital (Sigma, St Louis, MO, USA) and 50 μL sumianxin II (Jilin Shengda Animal Pharmaceutical Co., Ltd., Jilin, China) via intraperitoneal administration. Tropicamide-phenylephrine ophthalmic solution (Shenyang Xingji Corporation, Shenyang, Liaoning Province, China) was used to dilate the mouse pupil. ERG was recorded by full-field stimulation using a computer system (RETI port; Roland Consult GmbH, Brandenburg, Germany) equipped with international standards for visual electrophysiology (ISEVE) procedures. Moreover, a silver chloride electrode was placed at the centre of the cornea as the recode electrode. Stainless steel needle electrodes placed in the cheek and tail served as the reference and ground electrodes, respectively. We analysed the b wave (dark-adaptation 3.0 response) and dark-adaptation oscillatory potentials. Levofloxacin eye drops (Shenyang Xingji Corporation, Shenyang, Liaoning Province, China) were used three times a day after ERG testing to avoid infection.

### OCT, fundus photograph and FFA detection

The mice were anaesthetized as described before and corresponding optical coherence tomography (OCT) scans were performed using a Micron IV fundus camera and an OCT Scan Head equipped with a mouse objective lens. Right eyes were dilated with 0.5% tropicamide, and Gatifloxacin Eye Gel (Shenyang Xingji Corporation, Shenyang, Liaoning Province, China) was used to protect the corneas. Fundus and OCT images were captured from 20 positions for each eye using a Retinal Imaging System (OPTO-RIS, Optoprobe, Canada) and 4D-ISOCT Microscope Imaging System (ISOCT, Optoprobe, Canada). The original OCT data were analysed by using OCT Image Analysis software (Version 2.0, Optoprobe, Canada). Moreover, FFA was applied to detect retinal vessel microcirculation function by HRAplusII (Ger) after injecting 0.1 mL/100 g 10% fluorescence sodium (Baiyunshan Mingxing Corporation, Guangzhou, Guangdong Province, China) via intraperitoneal injection (Figure 4E). The sodium fluorescein intravenous imaging time was recorded to evaluate retinal vessel function.

### HE staining

Mice were euthanized with an overdose of sodium pentobarbital and the eyes of the mice were rapidly removed, and HE staining was implemented as we described previously [46]. Specifically, the eyes used for histological analysis were kept immersed for 24 h at 4° C in 4% paraformaldehyde. Three paraffin-embedded sections (thickness, 4 μm) were prepared and subjected to HE staining. Light microscope images were obtained using a digital imaging system (DP71, Olympus, Japan). The numbers of outer nuclear layer (ONL) and inner nuclear layer (INL) cell layers were then counted.

### Immunofluorescence staining

Eye paraffin sections were deparaffinized and dehydrated. Endogenous peroxidase activity was blocked with 3% H_2_O_2_ for 15 min followed by 3 washes with phosphate-buffered saline (PBS: 0.1 mM, pH 7.2) at room temperature every 5 minutes. Antigen was retrieved by boiling (100° C) in citric acid buffer (PH 6.0) for 20 min and addition of 10% goat serum (including 0.3% Triton) for 1 h at room temperature to block non-specific labelling. Then, the sections were incubated overnight at 4° C with primary antibody against GRP78 (Proteintech, 11587-1-AP, Wuhan, Hubei province) at a 1:200 dilution and ALDH2 (Abcam, ab108306, Cambridge, MA) at a 1:200 dilution. Slides incubated without any primary antibody served as the control. The slides were washed 3 times with PBS and incubated for 1 h with the IgG (H+L), Alexa Fluor 488 fluorescence secondary antibody (Zhuangzhi, EK021, Xi’an, Shaanxi province, China) at a 1:400 dilution. Nuclei were stained by incubating the sections in a 100 ng/mL DAPI after 3 rinses with PBS. Images of the slides were captured under a fluorescence microscope (BX53, Olympus, Japan).

### Western blot detection

Mice retinal tissues were separated and extracted on ice in RIPA buffer (Beyotime, Nantong, Jiangsu Province, China). The mixtures were then centrifuged at 12,000 rpm at 4° C for 15 min to collect the supernatant. A bicinchoninic acid (BCA) protein assay kit (Beyotime, Nantong, Jiangsu Province, China) was applied to calculate the concentration of the protein sample. An equal amount of protein was denatured by boiling with loading sample buffer followed by loading and separation of 25 μg protein by sodium dodecyl sulphate-polyacrylamide gel electrophoresis. Next, the protein was transferred onto a 0.22 μm PVDF membrane at 100 V for 90 min. The membranes were incubated with 5% non-fat milk solution (Sangon Biotech Co., Ltd, shanghai, China) for 1 h at room temperature and then reacted with ALDH2 (Abcam, ab108306, Cambridge, MA) at a 1:1000 dilution, eIF2α (Abcam, ab169528, Cambridge, MA) at a 1:500 dilution, phospho-eIF2α (Ser51) (Cell Signaling Technology, #3398, Danvers, MA) at a 1:1000 dilution, caspase9 (Cell Signaling Technology, D11A8, Danvers, MA) at a 1:500 dilution, caspase12 (Proteintech, 10380-1-AP, Wuhan, Hubei province, China) at a 1:1000 dilution, GRP78 (Proteintech, 11587-1-AP, Wuhan, Hubei province, China) at a 1:1000 dilution, and CHOP (Cell Signaling Technology, #5554, Danvers, MA) and GAPDH (Zhuangzhi Bioscience Technology Company, Xi’an, Shaanxi Province, China) at a 1:1000 dilution at 4° C overnight. The membranes were then incubated with the HRP-conjugated secondary antibody (Zhuangzhi Bioscience Technology Company, #EK020, Xi’an, Shaanxi Province, China) at a 1:10000 dilution at room temperature for 1 h, and then enhanced chemiluminescence was used for protein visualization. The intensity of immunoreactivity was quantified by densitometry using ImageJ software.

### ELISA

Retinal tissues were collected and interleukin-1 (IL-1), interleukin-6 (IL-6) and tumor necrosis factor-α (TNF-α) levels in retinas were estimated using commercially available enzyme-linked immunosorbent assay (ELISA) kit (Westang Bio-Tech Co., LTD, Shanghai, China) according to the manufacturer's instructions.

### Statistical analyses

Statistical analyses were performed using analysis of variance (ANOVA) followed by Bonferroni’s post hoc analysis to examine the differences among all groups. Quantitative data are presented as the mean ± standard deviation (SD), and *p*≤0.05 was considered statistically significant.

## Supplementary Material

Supplementary Figure 1
