# Supplementary figures and images for: ALDH2 protects naturally aged mouse retina via inhibiting oxidative stress-related apoptosis and enhancing unfolded protein response in endoplasmic reticulum
Source: Aging (Albany NY). 2020 Dec 19;13(2):2750–67. doi: 10.18632/aging.202325 (PMC7880320; doi:10.18632/aging.202325)

[www.aging-us.com](http://www.aging-us.com)

## AGING

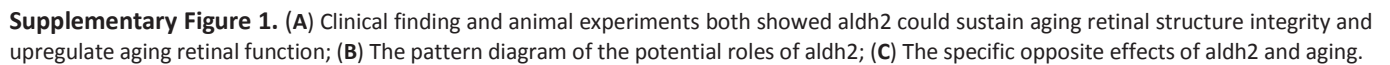

Supplement: Supplementary Figure 1 [file aging-13-202325-s001.pdf]
